# Challenges to the implementation of a multi-level intervention to reduce mistreatment of women during childbirth in Iran: a qualitative study using the Consolidated Framework for Implementation Research

**DOI:** 10.1186/s12978-024-01813-1

**Published:** 2024-05-27

**Authors:** Marjan Mirzania, Elham Shakibazadeh, Meghan A. Bohren, Sedigheh Hantoushzadeh, Abdoljavad Khajavi, Abbas Rahimi Foroushani

**Affiliations:** 1 Department of Health Education and Promotion, School of Public Health, Tehran University of Medical Sciences, Tehran, Iran; 2 Gender and Women’s Health Unit, Nossal Institute for Global Health, School of Population and Global Health, University of Melbourne, Carlton, VIC Australia; 3 Department of Obstetrics and Gynecology, School of Medicine, Vali-E-Asr Reproductive Health research Center, Family Health Research Institute, Tehran University of Medical Sciences, Tehran, Iran; 4 Department of Social Medicine, School of Medicine, Gonabad University of Medical Sciences, Gonabad, Iran; 5 Department of Epidemiology and Biostatistics, School of Public Health, Tehran University of Medical Sciences, Tehran, Iran

**Keywords:** Maternity care, Mistreatment, Multi-level intervention, Childbirth, CFIR, Implementation research, Qualitative study, Iran

## Abstract

**Background:**

Mistreatment during childbirth is a growing concern worldwide, especially in developing countries, such as Iran. In response, we launched a comprehensive implementation research (IR) project to reduce mistreatment during childbirth and enhance positive birth experiences in birth facilities. This study identified the challenges of implementing a multi-level intervention to reduce mistreatment of women during childbirth using the Consolidated Framework for Implementation Research (CFIR).

**Methods:**

An exploratory qualitative study, involving 30 in-depth interviews, was conducted between July 2022 and February 2023. Participants included a purposive sample of key stakeholders at different levels of the health system (macro: Ministry of Health and Medical Education; meso: universities of medical sciences and health services; and micro: hospitals) with sufficient knowledge, direct experience, and/or collaboration in the implementation of the studied interventions. Interviews were transcribed verbatim and coded using directed qualitative content analysis (CFIR constructs) in MAXQDA 18.

**Results:**

The identified challenges were: (1) individual level (childbirth preparation classes: e.g., adaptability, design quality and packaging, cosmopolitanism; presence of birth companions: e.g., patient needs and resources, structural characteristics, culture); (2) healthcare provider level (integrating respectful maternity care into in-service training: e.g., relative priority, access to knowledge and information, reflecting and evaluating); (3) hospital level (evaluating the performance of maternity healthcare providers: e.g., executing, external policies and incentives); and (4) national health system level (implementation of pain relief during childbirth guidelines: e.g., networks and communications, patient needs and resources, executing, reflecting and evaluating).

**Conclusions:**

This study provides a clear understanding of the challenges of implementing a multi-level intervention to reduce mistreatment of women during childbirth and highlights potential implications for policy makers and practitioners of maternal health programs. We encourage them to take the lessons learned from this study and revise their current programs and policies regarding the quality of maternity care by focusing on the identified challenges.

**Supplementary Information:**

The online version contains supplementary material available at 10.1186/s12978-024-01813-1.

## Abstract (plain language summary)

Evidence suggests that mistreatment during childbirth is a growing concern worldwide, especially in developing countries, such as Iran. In this qualitative study, through 30 in-depth interviews with key stakeholders at different levels of the health system (macro: Ministry of Health and Medical Education; meso: universities of medical sciences and health services; and micro: hospitals), we identified the challenges of implementing a multi-level intervention to reduce mistreatment of women during childbirth using the Consolidated Framework for Implementation Research (CFIR). The data were analyzed using directed content analysis and a deductive approach in MAXQDA 18 software. The identified challenges were: (1) individual level (childbirth preparation classes: e.g., adaptability; presence of birth companions: e.g., patient needs and resources); (2) healthcare provider level (integrating respectful maternity care into in-service training: e.g., relative priority); (3) hospital level (evaluating the performance of maternity healthcare providers: e.g., executing, external policies and incentives); and (4) national health system level (implementation of pain relief childbirth guidelines: e.g., networks and communications). This study provides a clear understanding of the challenges of implementing a multi-level intervention to reduce mistreatment of women during childbirth; and highlights potential implications for policy makers and practitioners of maternal health programs.

**Supplementary Information:**

The online version contains supplementary material available at 10.1186/s12978-024-01813-1.

## Background

Despite the recognition of every woman's right to enjoy the highest attainable standard of health, including the right to dignified and respectful care [1], evidence shows that mistreatment during childbirth is a common experience among women worldwide [2, 3]. It is increasingly recognized as an urgent public health priority and a poor quality of care index [1, 4], and is a critical determinant of women's decisions regarding place of birth, mode of birth, lactation, mother-child bonding, and childbirth experiences [5, 6]. The prevalence of mistreatment among women seeking maternity care varies across different settings, from 43% in Latin America and the Caribbean [7] to 76.3% in Europe (Germany and the Netherlands) [8]. The prevalence in Iran is likewise high, reported as 75.7% [9] and 100% [10]. Women in Iran have experienced verbal abuse, frequent and painful vaginal examinations, lack of continuity of care, empathy, participation in decision-making, choice of preferred birth position, privacy, and birth companions [11–14].

In recent years, some interventions have been developed, implemented, and showed promising results on reducing mistreatment and promoting respectful care for all women [15–18]. The Heshima project reported reductions in most forms of disrespect and abuse (D&A) in 13 health facilities in Kenya [15]. A study by Kujawski et al. (2017) in two hospitals in Tanzania (Staha project) showed a 66% reduction in the odds of women experiencing D&A after the intervention [17]. Asfa et al.'s (2020) study in Ethiopia showed that the intervention led to an 18% reduction in the number of mistreatment components [18].

In Iran, the Ministry of Health and Medical Education (MOHME) has developed a list of programs and practices to ensure maternal dignity during childbirth, such as the mother's bill of rights, maternal dignity training package, maternal dignity seminars for maternity healthcare providers (MHCPs) [19, 20], and emphasis on respectful maternity care (RMC) in the national guidelines for normal childbirth [21]. However, these actions did not make effective changes in the maternity quality of care. It seems that the programs implemented by the MOHME were not developed using context- and evidence-based approaches. There were also lacks of precise guidance on their effective implementation. Furthermore, the available research evidence on respectful/disrespectful maternity care in Iran has focused on the prevalence [9, 10], development and psychometrics of instruments [22, 23], and descriptions of women and healthcare providers’ experiences [24, 25], and few interventional studies have been conducted to reduce D&A or promote RMC, including workshops for midwives [26, 27]. It seems that healthcare providers training alone is not a sufficient solution [28]. In response, we launched a comprehensive implementation research (IR) project to reduce mistreatment during childbirth and enhance positive birth experiences in health facilities.

Prior to implementing any evidence-based intervention/innovation (EBI), it is important to identify the factors affecting its implementation in “real-world” settings to increase its adoption, scale-up, and sustainability [29]. It has been shown that many interventions that were effective in “in-vitro” and controlled conditions or small-scale fail in the real world due to contextual factors that acted against the implementation [30, 31]. Implementation science (IS) helps to identify factors that can support or inhibit implementation and to optimize intervention implementation. Therefore, although it is necessary to prove the effectiveness of interventions in trials, this is not sufficient to ensure successful implementation at scale. Therefore, it is necessary to understand why intervention works, how, for whom, and in what settings, and what strategies are needed to improve its implementation [32, 33].

In recent years, several models, theories, and implementation frameworks have been developed. The Consolidated Framework for Implementation Research (CFIR) [31] was developed by combining 19 theories on dissemination, innovation, implementation, organizational change, knowledge translation, and research uptake [34]. The CFIR is a “determinant framework” that consists of five domains, including the intervention characteristics (key features of an intervention), outer setting (features of the external context such as economic, political, and social environments of the intervention), inner setting (features of the organization such as structural, political, and cultural environments), characteristics of individuals involved (features of implementers such as cultural, organizational, and professional norms), and process of implementation (strategies or tactics that might influence the success of implementation) with 39 constructs/sub-constructs [34, 35] (Additional file 1: CFIR).

Despite attention to intervention studies to promote RMC or prevent mistreatment during childbirth, few studies have examined the implementation process of such interventions, and there is little insight into how the contextual conditions surrounding the implementation of these interventions contribute to their success or failure. To address this gap, we chose a qualitative method to obtain the experiences and perspectives of key stakeholders on the challenges of implementing a multi-level intervention to reduce mistreatment of women during childbirth in Iran using CFIR. Qualitative research methods are appropriate when seeking an in-depth understanding of participants' perspectives.

## Methods

This qualitative study was part of a larger implementation research project focusing on the development and implementation of a context-specific intervention to reduce disrespectful maternity care and evaluation of strategies to improve implementation. The project was initiated in October 2021 in five public teaching hospitals in Tehran, Iran, and consists of five phases: (1) needs assessment (to assess knowledge, attitudes and practices of maternity healthcare providers about mistreatment of women during labour and childbirth, and the manifestations of mistreatment and its influencing factors), (2) identifying interventions to reduce mistreatment of women during childbirth, (3) identifying the implementation challenges of interventions, (4) designing implementation strategies for the intervention, and (5) testing implementation strategies in a real-life setting. The findings of phase 1 of the project are described elsewhere [11, 36].

### Identifying interventions to reduce mistreatment of women during childbirth

**Fig. 1:**
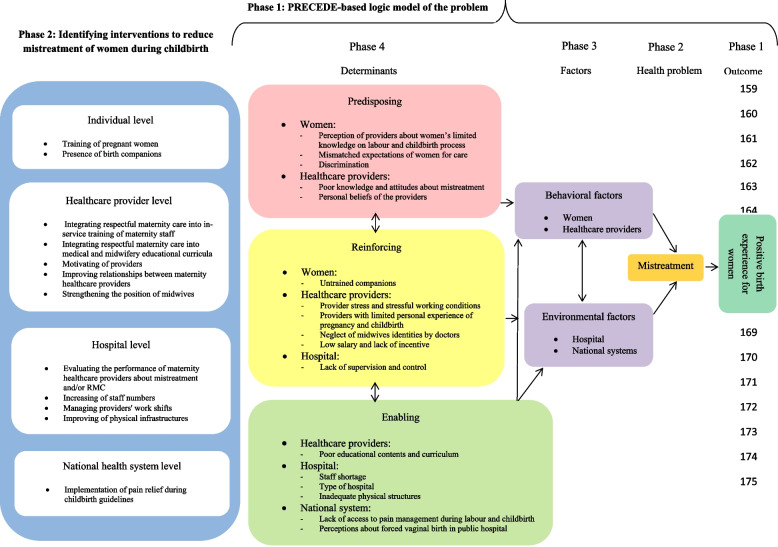
Logic model of the study

Based on the findings of phase 1 of the project, we created a logical model of the mistreatment problem based on the PRECEDE health-planning model [37]. According to the determinants of mistreatment based on the model, multi-level intervention was identified to address mistreatment drivers (Fig. 1). In this phase 3 of the project, we selected interventions from each level (individual, healthcare provider, hospital, and national health system) that are currently being implemented in Iran's health system to gain in-depth understanding of the challenges that affect proper implementation. Interventions implemented at each level are presented in Table 1. This study investigated the implementation challenges of these interventions.

**Table 1:** Interventions that are currently implemented in Iran’s healthcare system at individual, healthcare provider, hospital, and national health system level

Intervention	Description
Individual level	
Training of pregnant women	In Iran, childbirth preparation classes are currently implemented with the aim of empowering pregnant women in public hospitals (2008) and health centers (2014) as one of the strategies for promoting vaginal childbirth. However, evidence shows that women still generally have limited knowledge about labour and childbirth processes [11, 38], which can provide grounds for mistreatment [11, 39, 40].
Presence of birth companions	The possibility of presence of birth companions was provided by redesigning maternity wards to promote vaginal childbirth. However, most birth facilities do not provide birth companions [11, 25].
Healthcare provider level	
Integrating RMC into in-service training of maternity staff	In Iran, the first “congress on ethics in midwifery and reproductive health” with a focus on maternal dignity and the aim of increasing the knowledge, attitude, and behavioral intention of maternity hospital supervisors, was implemented at an online national workshop by the Ministry of Health and Medical Education (MOHME) in August 2022.
Hospital level	
Evaluating the performance of MHCPs about mistreatment and/or RMC	Iran’s Department of Midwifery (MOHME) developed a 27-item questionnaire to assess women's satisfaction with maternity services. This questionnaire was provided to the hospitals to be completed by the parturient women.
National health system level	
Implementation of pain relief during childbirth guidelines	The provision of childbirth pain relief methods is another strategy for promoting vaginal childbirth in Iran, which was implemented to make childbirth pleasant and to increase women's satisfaction. However, evidence shows that in public hospitals, pregnant women do not have access to pain relief options (mostly non-pharmacological) during labour [11, 41].

*RMC* Respectful Maternity Care, *MOHME* Ministry of Health and Medical Education, *MHCPs* Maternity Healthcare Providers

### Study design and participants

We conducted an exploratory qualitative study consisting of individual in-depth interviews between July 2022 and February 2023 in Tehran, Iran. Participants included key stakeholders at different levels of the health system (including healthcare providers, managers, experts, policy makers, and decision makers) with sufficient knowledge, direct experience, and/or collaboration in the implementation of each of the studied interventions. We selected participants using purposive sampling to obtain diverse perspectives and experiences and then used the snowball method to recruit more participants. We aimed for maximum variation among participants according to age, education, organizational role, and work experience. Key stakeholders were selected from three levels: macro (Ministry of Health and Medical Education (MOHME): four participants), meso (universities of medical sciences and health services: 12 participants), and micro (hospitals: 14 participants). These individuals were invited to participate by phone calls and/or in-person. The eligibility criteria for this study were familiarity and/or executive responsibility in any of the studied interventions and having at least five years of work experience.

### Data collection

We developed the initial semi-structured interview guide based on sample interviews at http://cfirguide.org [42]. Damschroder et al. (2009) recommend that researchers try to select constructs from CFIR that are most related to their study setting [34]. Therefore, the interview guide was revised using study-related constructs (Additional file 2: interview guide). We then pilot-tested this by conducting two initial interviews, which were not analyzed. Interviews were conducted in Persian by the lead author (M.M.), a female PhD candidate in Health Education and Promotion with previous experience in qualitative studies who had no prior interactions with the participants. To prepare participants for the interview, interview guide questions were sent to them in advance via email. Additionally, at the beginning of the interviews, the purpose of the study, guarantee of confidentiality and anonymity of information, nature of voluntary participation, and the possibility of withdrawing from the study at any time were explained to the participants. All participants provided written informed consent and permission for audio recordings. The interviews were conducted in participants' workplaces (in a private room) and during their preferred accommodation. The duration of the interviews ranged from 40 to 60 min, during which the interviewer made field notes. The demographic characteristics of the participants (including age, gender, education, organizational role, and number of years of work experience) were recorded at the end of each interview. The interviews continued until data saturation was reached. Saturation was obtained after the 28th interview; however, to ensure that no new information emerged, data collection continued until the 30th interview. All the invited individuals participated in the interviews, and no repeat interviews were conducted.

### Data analysis

Data analysis was conducted simultaneously with data collection, using directed content analysis [43] and a deductive approach. After each interview, M.M. listened to the recorded audios several times, transcribed verbatim in Persian, and returned to the participants for comments and/or corrections. E.Sh. (female professor in Health Education and Promotion; an experienced qualitative researcher) checked the transcripts for accuracy and consistency. Prior to coding the data, a categorization matrix was developed based on the interview guide (i.e., CFIR constructs). Next, two authors (M.M. and E.Sh.) independently analyzed the data. We marked and color-coded the significant segments of the text. We put those color-coded text segments together and gave codes. We categorized the codes according to their differences and similarities, and linked them to pre-specified categorizations in sub-themes and themes. If disagreements arose in coding, the authors discussed until consensus was reached. The MAXQDA 18 software was used to manage the data [44]. We translated selected quotes into English to support the themes developed throughout the analysis.

### Rigor

The trustworthiness of this study was tested based on the four criteria of Lincoln and Guba [45]. The credibility of the data was ensured through prolonged engagement with the data, applying a sampling technique with maximum variation, multiple data sources (including field notes, audio recordings, and transcripts), and providing initial codes to the three participants for approval. To enhance the transferability of the data, we conducted interviews with participants who had the most experience and knowledge of each of the studied interventions. Furthermore, dependability was obtained by analyzing the data separately by the two members of the research team. To assess confirmability, a qualitative research specialist, who did not participate in this study, confirmed the data analysis process. This paper was reported in accordance with the consolidated criteria for reporting qualitative research (COREQ) checklist [46] (Additional file 3: COREQ Checklist).

## Results

### Characteristics of participants

Thirty in-depth interviews were conducted with the key stakeholders. The mean age of the participants was 49.5 years (range: 35-65 years). Most participants (73.4%) held an MD or PhD degree. Four participants worked in MOHME, 12 in medical universities, and 14 in hospitals; more than half had over 20 years of work experience (Table 2).

**Table 2:** Demographic characteristics of the key stakeholder participants (*n*=30)

Characteristics	n	%
Age (years)		
< 40	6	20.0
40-49	9	30.0
50-59	11	36.7
60+	4	13.3
Mean ± SD = 49.5 ± 8.7		
Gender		
Female	25	83.3
Male	5	16.7
Education		
Ph.D	11	36.7
M.D	11	36.7
Master	4	13.3
Bachelor	4	13.3
Professional profile		
Obstetrician-Gynecologist	7	23.3
Midwife	5	16.7
Reproductive Health Specialist	7	23.3
Anesthesiologist	4	13.3
Health Policy Specialist	1	3.3
Health Services Management Specialist	1	3.3
Health Education and Promotion Specialist	1	3.3
Public Health- related manager	4	13.3
Organization		
Department of Maternal Health/ MOHME	3	10.0
Department of Education and Empowerment of Employees/ MOHME	1	3.3
Faculties/ Medical universities	6	20.0
In-service training center for health system employees/ Medical universities	4	13.3
Health Deputy and Treatment Deputy/ Medical universities	2	6.7
Hospital	14	46.7
Years of experience		
≤ 10	3	10.0
11-20	11	36.7
> 20	16	53.3

*SD* Standard Deviation, Organization: One interviewee might work in more than one organization, *MOHME* Ministry of Health and Medical Education

### The identified challenges

The challenges of implementing each intervention (currently implemented in the system) were identified and categorized using the domains and constructs/sub-constructs of the CFIR (Table 3).

**Table 3:** Challenges of implementation of a multi-level intervention to reduce mistreatment of women during childbirth; CFIR domains and constructs/ sub-constructs

CFIR domain	CFIR construct/ sub-construct	Interventions (Level/ name)				
		Individual		Healthcare provider	Hospital	National health system
		Childbirth preparation classes	Presence of birth companions	Integrating RMC into in-service training	Evaluating the performance of MHCPs about mistreatment and/or RMC	Implementation of pain relief during childbirth guidelines
Intervention characteristics	Adaptability	✓				
	Design quality and packaging	✓				
Outer setting	Patient needs and resources	✓	✓			✓
	Cosmopolitanism	✓				✓
	External policies and incentives			✓	✓	✓
Inner setting	Structural characteristics		✓			
	Networks and communications					✓
	Culture		✓			
	Compatibility		✓			
	Relative priority			✓		
	Organizational incentives and reward	✓				
	Available resources	✓		✓		✓
	Access to knowledge and information			✓		✓
Characteristics of individuals involved	Knowledge and beliefs about the intervention			✓		✓
	Other personal attributes	✓	✓			✓
Process of implementation	Executing	✓			✓	✓
	Reflecting and evaluating	✓	✓	✓		✓

*MHCPs* Maternity Healthcare Providers, *RMC* Respectful Maternity Care, The check mark in the cell indicates that there is a challenge

### Individual-level interventions

At the individual level, two interventions were listed according to the determinants of mistreatment based on the model: childbirth preparation classes and the presence of birth companions (Fig. 1). Both interventions are implemented in the system; however, there were serious challenges in the settings, as outline below.

#### Training of pregnant women about the process of labour and childbirth, respectful care and their rights during childbirth

In our study, participants shared opinions about the challenges of implementing childbirth preparation classes in five CFIR domains (intervention characteristics, outer setting, inner setting, characteristics of individuals involved, and process of implementation).

### Intervention characteristics

#### Adaptability

The level of adaptability of the intervention (childbirth preparation classes) was described as a key barrier to its implementation by most participants. They believed that non-compliance of the conditions and facilities of maternity hospitals with the educational content of the classes, improper timing of the start of classes (from the 20th week of pregnancy), and poor announcements can weaken the implementation of the intervention. The participants suggested that for effective childbirth preparation classes, the situations of facilities of maternity hospitals can be tailored and refined according to the educational content of the classes. Additionally, classes should be held in the early phases of pregnancy and widely announced.

*“The training that women receive in classes is different from that implemented in maternity hospitals. For example, we teach that they can move during labour, take their preferred position during childbirth, and have a chosen companion. However, in practice, this has not been implemented in maternity hospitals ...”* (Reproductive Health Specialist, University level)

*“Announcing about childbirth preparation classes in hospitals and health centers is poor. Only 18% of the pregnant women participated in classes. We did not announce them correctly…”* (Health Policy Specialist, MOHME level)

#### Design quality and packaging

Weakness in the design quality and packaging of childbirth preparation classes prevent their successful implementation. Some participants (obstetricians) reported a lack of a multidisciplinary team in holding classes as a barrier to implementation. They believed that classes should be managed by a team and should not be exclusive to midwives. However, the midwives stated that the content of the classes was such that it could be handled by them, but the presence of a psychologist in some sessions could play an important role in the success of the classes.

*“We must accept that midwives cannot cover all sessions. Psychologists, nutritionists, and obstetricians can be used in these classes.”* (Obstetrician, Hospital level)

### Outer setting

#### Patient needs and resources

Lack of training about RMC was also considered a fundamental factor. Most participants highlighted that women do not understand respectful care principles and their rights during childbirth, and this should be integrated into the content of childbirth preparation classes.

*“… They should be aware of their rights during childbirth. This should be integrated into the content of the childbirth preparation classes.”* (Obstetrician, MOHME level)

#### Cosmopolitanism

A crucial factor affecting childbirth preparation class implementation was the poor collaboration of the private sector to hold classes. Participants reported that since most pregnant women receive their care from the private sector (obstetricians and/or midwives' offices), there is a need to establish efficient mechanisms for more support and collaboration of these sectors in holding classes.

*“Participation of the private sector is essential because 70% of pregnant women receive their care from obstetricians and midwives.”* (Midwife, University level)

### Inner setting

#### Organizational incentives and rewards

A few participants expressed concerns about the poor implementation of childbirth preparation classes following the low participation of pregnant women in classes. They believed that setting enough incentives could affect women’s degree of engagement and commitment to participate in classes.

*“Between 9-10% of pregnant women attend our classes (health centers), and this rate is very low ... If incentives are provided, they are more motivated to participate.”* (Reproductive Health Specialist, University level)

#### Available resources

Participants reported that poor physical environment and staff shortages were barriers to implementing childbirth preparation classes.

*“In some hospitals, there is no standard space to hold classes, especially in private hospitals.”* (Midwife, Hospital level)

*“Dedicated instructors should be considered in these classes. Here, they appoint one person as an instructor, and at the same time, she has to work shifts in the maternity hospital because they do not have staff.”* (Midwife, Hospital level)

### Characteristics of individuals involved

#### Other personal attributes

Instructors’ skill and interest was another challenge that was highlighted by some participants: *“Unfortunately, some of our midwives (as instructors of classes) are rarely interested in training or do not have enough skills …”* (Reproductive Health Specialist, Hospital level)

### Process of implementation

#### Executing

Poor execution of childbirth preparation classes, especially during the COVID-19 pandemic, was an important challenge discussed by participants. They also believed that focusing on quantity and neglecting the quality of the classes made them not have the proper efficiency, and their goal was rarely reached: *“The classes are implemented, but they are not implemented according to plan and properly ... Unfortunately, we focused on the quantity of the classes, for example, the forms we have to complete and the statistics we have to give to the MOHME.”* (Reproductive Health Specialist, University level)

#### Reflecting and evaluating

Supervising implementation and continuous evaluation were crucial factors emphasized by the participants. They acknowledged that the MOHME should supervise the implementation of childbirth preparation classes in hospitals and health centers through regular inspections. In addition, evaluate the progress and quality of their implementation through an external evaluation.

*“I think the biggest challenge of childbirth preparation classes is that there is no supervision of their implementation … There should be a monitoring and auditing system.”* (Reproductive Health Specialist, MOHME level)

#### Presence of birth companions

In this study, the challenges of implementing birth companions in four CFIR domains (outer setting, inner setting, characteristics of individuals involved, and process of implementation) were discussed by the participants.

### Outer setting

#### Patient needs and resources

According to the participants, the lack of knowledge of companions could be a barrier to their attendance at maternity hospitals. Some participants believed that a person going to be a birth companion should be required to participate in childbirth preparation classes and receive training:

*“Companions have limited knowledge. I think birth companions should be required to participate in childbirth preparation classes because those who are trained in these classes are helpful to both labouring women and us providers.”* (Reproductive Health Specialist, Hospital level)

### Inner setting

#### Structural characteristics

The lack of physical space in some maternity hospitals was another factor that some participants stated: *“Some of our maternity hospitals do not have a standard structure, for example, Hospital X, which is a hall with 12 beds and set up some extra beds because of the high visits, so there will be no place for the presence of a birth companion.”* (Health Policy Specialist, MOHME level)

#### Culture

The participants also reported cultural issues as barriers to the implementation of birth companions. They noted that most of the time, if the companion is a partner, due to the feminine environment of maternity hospitals and female providers’ unwillingness to be accompanied by men in the delivery room; they are not allowed to be accompanied.

*“The companion is not allowed to enter the maternity hospital; why? Because my colleague (midwife or doctor) does not like a man to be in the labour room, she says, 'No, sir, you go out and let a woman come.”* (Reproductive Health Specialist, MOHME level)

#### Compatibility

One potential barrier to implementation was concern about the compatibility of the presence of birth companions with the existing workflows of maternity staff. The participants agreed that the interference of birth companions in the clinical duties of staff was a major factor for not allowing a companion.

*“As a midwife who worked in a maternity hospital for several years and was strongly against the presence of birth companions, I say that our main challenge was the interference of companions. For example, when a labouring woman's serum runs out, the companion comes many times and warns …”* (Midwife, University level)

### Characteristics of individuals involved

#### Other personal attributes

Some participants believed that the unwillingness of staff was an important barrier. They mentioned that staff prevents the presence of birth companions because of the perception that the companion is witnessing their performance as an advocate for the woman, which may cause them to expect more attention to labouring women.

*“The companion is like an advocate; it is like a hidden camera. Why do some staff members not like companions to enter maternity hospitals? This is because it controls their performance …”* (Obstetrician, Hospital level)

### Process of implementation

#### Reflecting and evaluating

Another factor was related to lack of supervision. The participants highlighted the need for continuous supervision of the implementation of birth companion guidelines in hospitals: *“The presence of birth companions has a guideline that has been communicated to all hospitals, but in many hospitals, especially public hospitals, it is not implemented because it is not supervising ...”* (Obstetrician, University level)

### Healthcare provider-level intervention

At this level, five interventions were listed according to the determinants of mistreatment based on the model (Fig. 1). However, one of them (integrating RMC into the in-service training of maternity staff) is implemented in the system. The challenges of this intervention were identified as follows:

#### Integrating RMC into in-service training of maternity staff

Participants in this study reported intervention implementation challenges in the four CFIR domains (outer setting, inner setting, characteristics of individuals involved, and process of implementation).

### Outer setting

#### External policies and incentives

Regulations and guidelines related to in-service training of staff affect the quality and efficiency of courses. Weakness in some regulations and guidelines has caused staff to be given a quantitative view, which means that many of them participate in the training course to obtain a certificate, rather than improve their knowledge, skills, and behavior, and/or increase the organization's productivity.

*“… Unfortunately, our regulations and guidelines are quantitative; that is, they dictate that if a person spends X hours in a year, it will be included in his/her evaluation and career promotion. Therefore, staff members only participate in courses to complete their duty hours and obtain a certificate.”* (Midwife, University level)

### Inner setting

#### Relative priority

Obtaining a license to hold an in-service training course was one of the challenges mentioned by some of the participants. They expressed the belief that the necessity of holding a respectful care training course should be clarified in the steering committee of training and empowerment of human resources in such a way that the course is included in the specialized and mandatory training of employees, not general and optional; thus, it is effective in their career development and they have sufficient motivation to participate in the course.

*“One of the challenges is to obtain a license to hold the course. You must justify the necessity of holding a respectful care training course in such a way that the course is included in the job description of the maternity staff.”* (Public Health- related manager, University level)

#### Available resources

Allocation of an insufficient budget for staff training was an important challenge reported by some participants. They found that staff participation in training courses required more financial support: *“Unfortunately, the investment in training staff is very low. The per capita education budget for healthcare staff training this year is 800,000 Iranian rials (IRR), is very small.”* (Public Health- related manager, MOHME level)

Similarly, the lack of experienced instructors is considered a challenge. When the instructor of an in-service training course does not have specialized knowledge and teaching ability, the course does not have the necessary efficiency and is not welcomed.

#### Access to knowledge and information

The participants also believed that informing the staff about the value and importance of the training course played an important role in its successful implementation. They highlighted that information and materials about the importance of RMC should already be provided to the maternity staff. A participant said: *“First, it clarifies the importance of respectful care training for the maternity staff. They need to know how much their behavior with labouring women can affect their mental health status as well as their decisions for future pregnancies.”* (Health Services Management Specialist, University level)

### Characteristics of individuals involved

#### Knowledge and beliefs about the intervention

Managers do not believe in in-service training for staff, and lack of support for them has caused the need for this training to not be included in the organization's plans and priorities.

*“Some managers do not support participation in training courses, and they do not believe that these courses have many benefits for the individual and organization.”* (Public Health- related manager, University level)

### Process of implementation

#### Reflecting and evaluating

Another major challenge was the weakness of evaluating the effectiveness of the training courses. The participants acknowledged that, although the evaluation of the effectiveness of courses is done using Kirkpatrick's model [47], it is often incomplete or limited to the first two levels of this model, and the third and fourth levels are not done because of problems and complexity.

*“... Our current evaluation method is flawed, and we do not evaluate almost any of our courses at the level of behavior; therefore, we cannot be sure if the person who participated in the course acquired the expected capabilities.”* (Reproductive Health Specialist, University level)

### Hospital-level intervention

At the hospital level, four interventions were listed based on the model (Fig. 1). Of these, the evaluation of the performance of MHCPs is implemented in the system. The identified challenges for this intervention were as follows:

#### Evaluating the performance of MHCPs about mistreatment and/or RMC

In our study, participants discussed the intervention implementation challenges in two CFIR domains (outer setting and process of implementation).

### Outer setting

#### External policies and incentives

Some participants complained of weakness in laws and regulations. They stated that to supervise the performance of MHCPs in laws and regulations (including the Support of Family and Youth Population Act), the merit pay of providers dependent on the satisfaction of pregnant women is defined. However, they are not included in the payment systems of all MHCPs. Furthermore, participants expressed concern that these laws (such as reducing merit pay or warnings) were not very effective in supervising the performance of the providers.

*“Currently, in the Support of Family and Youth Population Act, merit pay of the providers depends on the satisfaction of pregnant women, but unfortunately not for all providers (including obstetricians or residents). We are pursuing this to be modified.”* (Health Policy Specialist, MOHME level)

### Process of implementation

#### Executing

Poor execution of the intervention (mother’s satisfaction questionnaire) was considered important. Participants stated that, although all hospitals were required to implement and provide feedback to the MOHME, the providers often completed the questionnaire. To solve this problem, an electronic satisfaction questionnaire is currently being designed, whose links will be sent to women, and their satisfaction reports will be registered in the Ministry of Health's portal. However, owing to the poor support of the Information Technology (IT) unit, it has not yet been implemented.

*“… Unfortunately, the questionnaires were completed by the providers, without the mother being informed. Currently, an electronic questionnaire is designed, the report of which will be registered in the Ministry of Health's portal, but it has not yet been implemented.”* (Reproductive Health Specialist, Hospital level)

### National health system-level intervention

At the national health system level, the implementation of pain relief during childbirth guidelines was listed based on the model (Fig. 1). This intervention is implemented in the system, and its challenges were as follows:

#### Implementation of pain relief during childbirth guidelines

In this study, the participants identified implementation challenges in the four CFIR domains (outer setting, inner setting, characteristics of individuals involved, and process of implementation).

### Outer setting

#### Patient needs and resources

Participants mentioned the lack of knowledge of pregnant women as an important challenge in implementing pain relief during childbirth. They believed that most women are unaware of the option of pain relief during childbirth. Pregnancy is an important time to inform and prepare women about pain relief options during childbirth; however, women are unaware of this right and do not demand it.

*“… Pregnant women do not have sufficient information regarding pain relief during childbirth … so they do not demand ... Information about this should be provided during pregnancy (for example, in childbirth preparation classes), but when labouring women come to the maternity hospital, we have to go and explain … I think that this is not the right time for training.”* (Anesthesiologist, Hospital level)

Some participants also pointed out that a large number of their clients are Afghan women who refuse pain relief, because they do not have insurance coverage and would be required to pay out-of-pocket.

*“... Most of our clients are Afghan women. They do not have insurance and have to pay for it. Therefore, they do not do (pain relief during childbirth).”* (Obstetrician, Hospital level)

#### Cosmopolitanism

The presence of good networking and relationships with external organizations, such as insurance organizations, to modify pain relief during childbirth tariffs and motivate staff was described by participants as an effective factor in implementation.

*“The support of insurance organizations is also crucial for the implementation of pain relief during childbirth; tariffs should be revised, but unfortunately, they do not collaborate.”* (Obstetrician, MOHME level)

#### External policies and incentives

MOHME policies and support were critical for the successful implementation of pain relief during childbirth. Some participants believed that being free of charge for pain relief during childbirth in public hospitals was one of the factors facilitating its implementation. However, the participants reported that some measures of the MOHME, including the absence of on-call anesthesiologists in hospitals were another challenge for the implementation of the program.

*“... The hospital should have an on-call anesthetist, which unfortunately the MOHME took it away ... Therefore; we do not have the possibility of pain relief during childbirth at night because we there is not have an on-call anesthesiologist. There is an aesthesia resident, but it is normal that she/he does not spend X hours on pain relief during childbirth and quickly performs a caesarean section.”* (Obstetrician, Hospital level)

### Inner setting

#### Networks and communications

Poor working relationships between obstetricians and anesthesiologists were key barriers. Some participants believed that obstetricians are the primary decision-makers for pain relief during childbirth, and if they approve, labouring woman will be referred to anesthesiologists, but unfortunately, they do not collaborate enough in this regard. A participant stated:

*“Obstetricians should select labouring women based on the criteria and then refer to them. Unfortunately, they do not collaborate with us …”* (Anesthesiologist, Hospital level)

#### Available resources

The availability of resources during the implementation process was critical for success. The participants complained about the low tariff allocated to pain relief during childbirth and considered it a fundamental barrier to non-collaboration of anesthesiologists in the implementation of the program. Furthermore, the lack of staff (anesthesiologists and nurse anesthetists) to offer top-ups and continuous monitoring adds to this factor.

*“Pain relief during childbirth is a time-consuming process, but the tariff is so low that the anesthesiologist does not want to perform it. However, there is a shortage of anesthesiologists and nurse anesthetists in most hospitals.”* (Anesthesiologist, Hospital level)

#### Access to knowledge and information

Similarly, limited access to knowledge and information about pain relief during childbirth for the provider team was considered another challenge. The participants identified a lack of adequate training for providers prior to implementing the program as a contributing factor.

*“Prior to the implementation of this program (pain relief during childbirth), sufficient training should have been provided to all team members (including anesthesiologists, obstetricians, and midwifes), and the purpose and importance of the program were well introduced. We were not justified at all as to why we wanted to do this program ...”* (Obstetrician, Hospital level)

### Characteristics of individuals involved

#### Knowledge and beliefs about the intervention

Another challenge was the lack of knowledge and misconceptions of providers (obstetricians and midwives) regarding pain relief during childbirth. For example, it is believed that pain relief during childbirth is associated with an increased risk of prolonged labour, poor maternal and infant outcomes, and an increased chance of cesarean section. The participants believed that there was a serious need to spread awareness and cultivate a positive attitude among providers about the benefits of pain relief during childbirth and eliminate misconceptions by holding training courses.

*“I think the most important challenge is misconceptions. Still, many obstetricians do not agree with pain relief during childbirth; it is believed that it prolongs the labour process or may have complications for the mother and/or the infant; all this is due to lack of knowledge. This belief needs to be corrected.”* (Anesthesiologist, Hospital level)

#### Other personal attributes

According to most participants, the lack of expertise and skills of anesthesiologists was another barrier to implementation. They acknowledged that pain relief during childbirth is one of the important abilities that anesthesia residents should acquire, which has not been considered in their educational curriculum. Anesthesiology residents spend a short period of one month in the maternity ward, so they do not acquire enough skills.

*“Pain relief during childbirth requires expertise and skill ... However, it has not been considered an important topic in the educational curriculum of anesthesiologists.”* (Midwife, University level)

### Process of implementation

#### Executing

Some participants felt that pain relief during childbirth had not been implemented according to the implementation plan. They emphasized the identification of program problems and the importance of proper planning: *“At first, the process of pain relief during childbirth in our hospitals was increasing; for example, in our hospital, we had about 500 pain relief during childbirths per month, but currently we do not have four ... We were weak in execution; we have implemented the program since 2014, but unfortunately, I can say that we have been unsuccessful thus far.”* (Anesthesiologist, Hospital level)

#### Reflecting and evaluating

In addition, supervising the implementation of pain relief during childbirth in hospitals was another factor mentioned by some participants. They stated that internal and external inspections should be used to supervise the performance of the team in providing pain relief during childbirth.

*“There must be supervision ... If it is not done (pain relief during childbirth), it is not supervised that why was it not done? The mother did not request or you (providers) did not?”* (Health Education and Promotion Specialist, University level)

## Discussion

In this study, using the CFIR, we identified perspectives of key stakeholders from different levels of the health system regarding the challenges of implementing a multi-level intervention to reduce mistreatment of women during childbirth in Iran. Overall, the findings showed that through the lens of CFIR domains (intervention characteristics, outer setting, inner setting, characteristics of individuals involved, and process of implementation), there are several challenges to successfully implementing current interventions. Documenting the findings of such studies can help formulate appropriate strategies to improve the implementation of interventions to reduce mistreatment during childbirth as well as the development of high-quality maternity care guidelines in similar settings.

In our study, the most identified challenges to successfully implementing the interventions were related to the outer- and inner-setting domains. The key role of the outer setting [48, 49] and inner setting [50], which emphasize the external influences on the intervention and characteristics of the implementing organization, has been highlighted in other studies for successful implementation. Our findings showed that all proposed interventions were influenced by factors from the outer setting. Patients’ needs and resources could challenge the implementation of childbirth preparation classes, birth companionship, and pain relief during childbirth. Participants reported that women were not trained in childbirth preparation classes about respectful care principles and their rights during childbirth, birth companions were not trained, and most women were unaware of pain relief during childbirth. Previous studies are in agreement with our findings and reflect the need for respectful care education for women [51, 52], the presence of a trained birth companion [25, 53], and training programs to increase women's awareness of pain relief options during childbirth [54]. In this study, poor collaboration with external organizations was also identified as a barrier to the implementation of childbirth preparation classes and pain relief during childbirth. The Heshima project in Kenya showed that participatory design of interventions at the policy, facility, and community levels played a significant role in the public acceptance of maternity care and health rights; therefore, the successful implementation and sustainability of the RMC intervention requires the formation of partnerships with external organizations [55]. Furthermore, our findings showed that external policies (including weakness in regulations and guidelines) challenge the implementation of interventions (including integrating RMC into in-service training, evaluating the performance of MHCPs, and pain relief during childbirth). Similar to our findings, Warren et al. (2017) reported that the free maternity care policy in Kenya affected the quality of care by increasing the demand for health facilities, delays in financing, augmented provider workloads and shortages, and posed challenges to the implementation of RMC [55]. In another study (2021), the existing policy in the West Bank to prevent the presence of birth companions in public facilities was reported by participants as a factor for mistreatment during childbirth [56].

Inner setting factors that affected the implementation of interventions were structural characteristics, networks and communications, culture, compatibility, relative priority, organizational incentives and rewards, and readiness for implementation (available resources and access to knowledge and information). Participants acknowledged that structural characteristics (including a lack of physical space), cultural issues, and incompatibility can act as barriers to birth companion intervention. Studies support the findings of our study that the limitations of the physical structure of hospitals make it difficult to allow birth companions [28, 57]. In addition, in our study, as in some cultures, the presence of male partners was not socially acceptable, especially during childbirth [58], and there were concerns about the interference of birth companions in healthcare providers’ medical decisions [58]. Moreover, in this study, participants stressed the importance of holding a respectful care training course for providers. Healthcare providers have been shown to have a negative attitude toward respectful care [59], which is often less important than other aspects of care [60]. Poor working relationships between providers were another factor affecting implementation. Similarly, poor teamwork among obstetricians, midwives, and anesthesiologists was highlighted as an important barrier to implementing labour analgesia in Wu et al.'s study [61]. Moreover, in our study, lack of resources (including physical space, human resources, money, and training) was described as a potential barrier to readiness for implementation. In addition, limited access to knowledge and information about the intervention was another barrier to readiness for implementation. Our findings are consistent with previous studies that have examined how inner setting characteristics such as readiness for implementation [55] and organizational rewards and incentives [62] influence implementation.

The intervention characteristics domain, which emphasizes the importance of the need to adapt the intervention to enhance its fit with the context [63], is a critical determinant of the success of implementation [34, 64, 65]. In our study, adaptability and design quality and packaging were seen as important factors in the implementation of childbirth preparation classes. The findings showed that the situations of facilities of maternity hospitals do not adapt the content of the classes, the timing of the start of the classes are not appropriate, and they are not announced correctly. Previous studies have assessed the factors influencing childbirth preparation classes; for example, a study conducted by Otogara (2017) reported the need for the presence of a psychologist consultant as well as appropriate timing and information for successful implementation of classes [66].

The domain of the characteristics of individuals involved in the intervention is also crucial to ensure the success of implementation [34]. Our findings showed that the personal attributes of individuals within the organization (such as interest, skills, and expertise), as well as their knowledge and beliefs about the intervention, are other key factors that can hinder implementation. Other studies have similarly shown that competent, skilled, and motivated service providers are important for RMC provision [67]. Moreover, providers' knowledge and understanding of RMC are reported to be important in designing interventions to address mistreatment in maternity care [68]. In Mexico, training and enabling healthcare providers to promote respectful delivery care have been noted [69].

In our study, the implementation process domain was identified as a key factor in the implementation of all the studied interventions. Participants noted suboptimal execution, lack of supervision, and weakness in evaluating posed challenges for implementing interventions. Previous studies have revealed the key role of monitoring and evaluation interventions in the success of RMC implementation [15, 70]. This was implemented in the Hashima project by applying mechanisms to report cases of disrespect, such as customer service desks, suggestion boxes and supervisory visits at the facility level [15].

Overall, our study has potential implications for practice and research. This study highlights practical benefits for policy makers and practitioners of maternal health programs in Iran and other contexts. We suggest that they consider the findings of this study when implementing their current programs and policies regarding the quality of maternity care. Moreover, intervention studies focusing on RMC and/or mistreatment during childbirth appear to be relatively limited in high-income countries (HICs), and research and implementation efforts in these settings must continue. The implementation process of these interventions has been inadequately explored, thus affecting their comparability. Using the CFIR, this study provides important insights into how the contextual conditions surrounding the implementation of multi-level interventions to reduce mistreatment during childbirth contribute to their success or failure. Therefore, the findings of this study can provide evidence for formulating effective strategies with the potential to increase the positive experiences of childbirth for women.

### Strengths and limitations

To the best of our knowledge, this is the first attempt to identify the challenges of implementing a multi-level intervention to reduce mistreatment of women during childbirth in Iran and provides important insights into the contextual conditions around the implementation of each of the interventions. Our findings can be useful for other developing countries (LMICs) in similar contexts, especially those in the Eastern Mediterranean region. Reflecting the perspectives of key stakeholders from the micro- to macro-level of the health system was another strength of our study. Furthermore, the use of CFIR as the most common framework in IS allowed us to comprehensively identify the effective factors in the implementation of each intervention. However, given that the interviews were conducted with key stakeholders involved in the interventions, there is a possibility of a social desirability bias (underreporting of actual experiences and challenges due to their roles). We tried to mitigate this limitation by guaranteeing the confidentiality and anonymity of information as well as, conducting interviews in a private room. Also, this study focused on the interventions that are currently implemented in Iran's health system; and further research is needed to explore the implementation challenges of other interventions intended to reduce mistreatment during childbirth.

## Conclusions

Our findings revealed potential challenges for implementing a multi-level intervention to reduce mistreatment of women during childbirth in the domains of intervention characteristics, outer setting, inner setting, characteristics of individuals involved, and process of implementation of the CFIR. Addressing these challenges is necessary to improve the implementation of interventions to reduce mistreatment during childbirth in Iran.

### Supplementary Information

Supplementary Material 1.

Supplementary Material 2.

Supplementary Material 3.

## Data Availability

The datasets generated and analyzed during the current study are not publicly available due to privacy restrictions of the participants but are available from the corresponding author on reasonable request.

## Abbreviations

D&A: Disrespect and Abuse
RMC: Respectful Maternity Care
EBI: Evidence-Based Intervention/Innovation
IS: Implementation Science
CFIR: Consolidated Framework for Implementation Research
MOHME: Ministry of Health and Medical Education
MHCPs: Maternity Healthcare Providers
COREQ: Consolidated Criteria for Reporting Qualitative Research
IRR: Iranian Rials
IT: Information Technology
HICs: High-Income Countries
LMICs: Low and Middle Income Countries
