# Supplementary material for: Challenges to the implementation of a multi-level intervention to reduce mistreatment of women during childbirth in Iran: a qualitative study using the Consolidated Framework for Implementation Research
Source: Reprod Health. 2024 May 27;21:70. doi: 10.1186/s12978-024-01813-1 (PMC11131232; doi:10.1186/s12978-024-01813-1)
Supplement: Supplementary file 2 — Supplementary Material 2. [file 12978_2024_1813_MOESM2_ESM.docx]

**Additional file 2: Interview Guide**

- Please explain how the intervention was developed and implemented.
- How did hospitals become involved with the intervention?
- What was your impression of the implementation of the intervention during this period?
- Do you think the intervention was effective or not?
- What kind of information or evidence do you know shows that the intervention was effective?
- In your opinion, how complicated is the implementation of the intervention?
- What were most hard parts to implement? Why?
- What are the barriers and/or challenges of implementing the intervention? Please explain.
- How did you overcome these barriers?
- In your opinion, what kind of change or changes should be made to the intervention for it to work effectively?
- How well does the implementation of this intervention meet the needs of patients (pregnant women)?
- What barriers will the people your organization serves (pregnant women) face in participating in the intervention?
- What incentives are provided to ensure the successful implementation of this intervention?
- What resources were required to implement the intervention? Were these resources available?
- Can you describe the planning you did to implement the intervention?
- How did you follow-up on what needed to be done? Progress? Situation?
- Has the intervention been implemented according to the plan? Please explain.
- At the end, is there anything we should talk about, or is any additional information that we have not discussed?
